# Supplementary material for: Review of Experimental Studies to Improve Radiotherapy Response in Bladder Cancer: Comments and Perspectives
Source: Cancers (Basel). 2020 Dec 30;13(1):87. doi: 10.3390/cancers13010087 (PMC7795454; doi:10.3390/cancers13010087)
Supplement: Supplementary file 1 [file cancers-13-00087-s001.pdf]

Table S1. Clinical trials of concurrent chemoradiotherapy in bladder cancer.

Study	Patient number and characteristics	Study design	Outcome	Ref.
Shipley *et al.*, 1987	70 T2-T4 MIBC patients	RT + cisplatin on a multi-institutional prospective protocol.	Complete response rate: 77% in the 62 patients completing planned irradiation and 70% for all patients.	[1]
Housset *et al.*, 1993	54 T2 -T4 MIBC patients	TUR + 5-FU-cisplatin combination + RT. A control cystoscopy was performed 6 weeks after completion of the neoadjuvant program. Patients with persistent tumor underwent cystectomy. Complete responders were treated by either additional CRT (group A) or cystectomy (group B).	At control cystoscopy, 74% had a histologically documented complete response. Metastatic disease in 16 patients, more frequently in the nonresponders (71%) than in responders (15%). Disease-free survival rate at 3 years: 62%.	[2]
Coppin *et al.*, 1996	99 T2-T4b N0 MIBC patients	Fractionated RT + intravenous cisplatin 100 mg/m^2 at 2-week intervals for three cycles vs RT alone.	Complete response: 47 vs 31%. 3-year overall survival 47 vs 33%; 5-year locoregional relapse rate 40 vs 59% ($p = 0.04$); bladder preservation 70 vs 36%.	[3]
James *et al.*, 2012.	360 MIBC patients	RT with or without synchronous chemotherapy. 5-FU (500 mg/mer day) during fractions 1 to 5 and 16 to 20 of RT+ mitomycin C (12 mg/m^2) on day 1.	2 year locoregional disease–free survival: 67% in the CRT group and 54% in the RT group. 5-year OS: 48% in the CRT group and 35% in the RT.	[4]
Thompson *et al.*, 2017	78 patients with MIBC	Concurrent CRT with gemcitabine (GemX) with or without neoadjuvant chemotherapy (neoGemX).	Only 49 patients included. No significant difference between mean scores at baseline and 12 months after treatment completion or between the neoGemX and GemX groups.	[6]

Hoskin *et al.*, 2010.	333 patients with locally advanced bladder carcinoma	RT alone versus RT with carbogen and nicotinamide (CON). The primary end point was cystoscopic control at 6 months (CC_{6m}) and secondary end points were overall survival (OS), local relapse-free survival (RFS), urinary and rectal morbidity.	CC_{6m} : 81% for RT + CON and 76% for RT alone (P .3); however, just more than half of patients underwent cystoscopy at that time. In multivariate analysis: RT CON significantly reduced risk of relapse (P .05) and death (P .03); no differences in late urinary or GI morbidity .	[7]
Murthy *et al.*, 2016	44 patients with localized bladder cancer.	TURBT + concurrent platinum based CT receiving either prophylactic nodal RT or escalated dose to the tumour bed.	Locoregional control (87% vs 68%, p= 0.748) and overall survival (74% vs 60%, p=0.36) were better in patients receiving dose escalation.	[22]
Efstathiou *et al.*, 2012	348 MIBC patients with T2-4a	Concurrent cisplatin-based CCRT after TURBT plus neoadjuvant or adjuvant chemotherapy. Repeat biopsy after 40 Gy, initial tumor response guiding subsequent therapy: CRT boost or cystectomy.	Median follow-up: 7.7 yr. Multivariate analyses: CR (72%) significantly associated with improved DSS and OS. No patient required cystectomy for treatment-related toxicity.	[34]

Table S2. Characteristics of BCa cell lines used in RT studies.

Cell line	Cellosaurus accession nr	*In vitro*	*In vivo*	Gender	Age	Grade	Stage	Ras Mut	p53 Mut	TERT-promoter Mut	FGFR3 gene alteration	Ref.	Part of UBC-40[1]	Part of CCLE[2]	Molecular classification [47]	Molecular classification [46]
**T24**	CVCL_0554	39	4	F	82Y	G3	pTa	X	X	X	wt	[121]	X	X	basal	non-luminal, non-basal
**RT112**	CVCL_1670	25	4	F	n/a	G2	pTa	wt	X	m/r	m/r	[122]	X	X	luminal	luminal
**5637**	CVCL_0126	11	3	M	68Y	G2	n/a	wt	X	wt	wt	[123]	X	X	mixed	basal
**RT4[3]**	CVCL_0036	8	2	M	63Y	G1-2	n/a[3]	wt	X	X	m/r	[124]	X	X	luminal	luminal
**UMUC3**	CVCL_1783	7	2	M	n/a	n/a	pT2-T4	X	X	X	wt	[125]	X	X	basal	non-luminal, non-basal
**HT1376**	CVCL_1292	5	0	F	58Y	G3	>= pT2	wt	X	X	wt	[126]	X	X	mixed	basal
**647V**	CVCL_1049	4	0	M	59Y	G2	pT2/3a	wt	X	X	m/r	[127]	-	X	basal	basal
**J82**	CVCL_0359	3	1	M	58Y	G3	pT3	wt	X	X	m/r	[128]	X	X	basal	non-luminal, non-basal
**HT1197**	CVCL_1291	3	0	M	44Y	G4	pT2	wt	m/r	X	X	[126]	X	X	mixed	basal
**KK47**	CVCL_8253	1	1	M	50Y	G1	n/a	wt	wt	wt	wt	[129]	X	-	n/a	non-luminal, non-basal
**SW780**	CVCL_1728	1	1	F	80Y	G1	n/a	wt	wt	X	m/r	[130]	X	X	luminal	luminal
**CAL29**	CVCL_1808	2	0	F	80Y	G4	pT2	wt	X	X	wt	[131]	-	X	luminal	n/c
**253J B-V**	CVCL_7937	2	0	M	53Y	G4	pT4	wt	wt	m/r	wt	[132]	X	X	basal	non-luminal, non-basal
**NTUB1**	CVCL_RW29	1	1	F	70Y	n/a	n/a	wt	wt	wt	wt	[133]	-	-	n/a	n/a
**UCRU-BL13**	CVCL_M873	1	0	M	62Y	n/a	n/a	wt	wt	wt	wt	[134]	-	-	n/a	n/a
**UCRU-BL17**	CVCL_M007	1	0	F	69Y	n/a	n/a	wt	X	wt	wt	[135]	-	-	n/a	n/a
**UCRU-BL28**	CVCL_4904	1	0	M	62Y	n/a	n/a	wt	X	wt	wt	[136]	-	-	n/a	n/a
**TCC-SUP**	CVCL_1738	2	0	F	67Y	G4	n/a	wt	X	X	wt	[137]	X	X	basal	non-luminal, non-basal
**UMUC5**	CVCL_2750	1	0	F	n/a	n/a	n/a	wt	X	X	wt	[138]	X	-	basal-like	luminal
**VMCUB1**	CVCL_1786	1	0	M	n/a	G2	n/a	wt	X	X	wt	[139]	X	X	basal	basal
**KU19-19**	CVCL_1344	1	0	M	76Y	n/a	n/a	n/a	X	X	wt	[139]	-	X	n/a	basal
**UMUC6**	CVCL_2751	1	0	M	n/a	n/a	n/a		X	n/a	wt	[125]	X	-	mixed	basal
**UMUC9**	CVCL_2753	1	0	M	n/a	n/a	n/a	n/a	X	X	n/a	[140]	X	-	Non-basal like	luminal
**SW-800**	CVCL_A684	1	0	F	n/a	n/a	n/a	n/a	X	X	n/a	[141]	X	-	Non-basal like	n/a
**639-V**	CVCL_1048	1	0	F	n/a	n/a	n/a	n/a	X	X	n/a	[127]	X		Basal-like	non-luminal, non-basal
MB49	CVCL_7076	*3*	*0*	*M*	*n/a*	*n/a*	*n/a*	*n/a*	*n/a*	*n/a*	*n/a*	[142]	-	-	Mouse BCa cell lines	
MB49-I	CVCL_VL62	*1*	*0*	*M*	*n/a*	*n/a*	*n/a*	*n/a*	*n/a*	*n/a*	*n/a*	[143]	-	-		

MBT2	*CVCL_4660*	*3*	*2*	*F*	*n/a*	*n/a*	*n/a*	*n/a*	*n/a*	*n/a*	*n/a*	[144]	-	-	

[1] UBC-40, a comprehensive genomic characterization of 40 urothelial bladder carcinoma (UBC) cell lines [47]; [2] CCLE, Cancer Cell Line Encyclopedia [145]**;** [3] RT4 cell line originates from re-occurring human transitional cell papilloma [124] *italic*: mouse BCa cell lines; abbreviations: n/a, information not available; n/c, not categorized (not coherent classification depending on the dataset used); m/r, mixed reports; wt, wild type; [3]Information from the https://web.expasy.org/. Abbreviations: TKI, tyrosine kinase inhibitor; n/a, information not available. Information in this table has been collected and updated of that from Zuiverloon *et al*. [48].

Table S3. Characteristics of problematic human BCa cell lines used in experimental RT studies.

Cell line	Cellosaurus accession no.	*In vitro*	*In vivo*	*Comment* [1]
MGHU-1 (EJ138)	CVCL_2443	8	0	T24 derivative
S40b	n/a	4	0	Parent cell line (MGHU-1) has been shown to be a T24 derivative
ECV304	CVCL_2029	3	0	T24 derivative
EJ30	CVCL_2443	3	0	Parent cell line (Ej138) has been shown to be a T24 derivative.
biu87	CVCL_6881	3	0	Contaminated by non-human cell
KU7	CVCL_4714	1	1	HeLa derivative
MGHU-2	CVCL_9826	1	0	T24 derivative
TSGH 8301	CVCL_A342	1	1	Contaminated by a cervix cancer cell line ME-180
TSU-Pr1	CVCL_4014	0	1	Problematic cell line: Contaminated. Shown to be a T24 derivative

[1] Information from the https://web.expasy.org/.

Table S4. Preclinical studies of problematic cell lines used in vivo.

Cell line	Subtype	IR regimen	Strategy of radiosensitisatio n	Sex	Mouse background	Initial tumour (mm^3) [1]	Study follow-up (days) [2]	Ref.
**TSGH 8301**	Problematic cell line: Contaminated by a cervix cancer cell line ME-180[3]	1 x 10 Gy	Mullberry Water Extract	M	BALB/c nude	140	21	[146]
**TSU-Pr1**	Problematic cell line: Contaminated. Shown to be a T24 derivative[3]	*1 x 5Gy*	Adenoviral vector-mediated GLIPR1 gene therapy	*M*	BALB/c nude	100	*72*	[147]
KU7	Problematic cell line: Contaminated. Shown to be a HeLa derivative[3]	*3 x 3Gy*	mTOR inhibitor	*F*	Athymic Nu/Nu	Not mentioned	*?*	[148]

[1] The initial size of the tumour is defined as the size of the tumour at the start of the RT or combination treatment (Day 1); [2] The minimum follow-up for the non-treated control were used to compare the growth of the xenografts; [3] Information from the https://web.expasy.org/.
